# Learning to Select Supplier Portfolios for Service Supply Chain

**DOI:** 10.1371/journal.pone.0155672

**Published:** 2016-05-19

**Authors:** Rui Zhang, Jingfei Li, Shaoyu Wu, Dabin Meng

**Affiliations:** 1 College of Management and Economics, Tianjin University, Tianjin, China; 2 School of Computer Science and Technology, Hulunbuir College, Hulunbuir, China; 3 School of Computer Science and Technology, Tianjin University, Tianjin, China; 4 School of Economics, Tianjin University of Commerce, Tianjin, China; Southwest University, CHINA

## Abstract

The research on service supply chain has attracted more and more focus from both academia and industrial community. In a service supply chain, the selection of supplier portfolio is an important and difficult problem due to the fact that a supplier portfolio may include multiple suppliers from a variety of fields. To address this problem, we propose a novel supplier portfolio selection method based on a well known machine learning approach, i.e., Ranking Neural Network (RankNet). In the proposed method, we regard the problem of supplier portfolio selection as a ranking problem, which integrates a large scale of decision making features into a ranking neural network. Extensive simulation experiments are conducted, which demonstrate the feasibility and effectiveness of the proposed method. The proposed supplier portfolio selection model can be applied in a real corporation easily in the future.

## Introduction

In the environment of economic globalization and knowledge economy, more and more manufacturers shift their management model from product provision to the provision of service solutions [32]. For example, Hong Kong’s Fung group (URL: http://www.funggroup.com/eng/global/home.php) created as an import and export trading company at 1906, then gradually transformed into a global provider of integrated supply chain solutions. IBM (URL: http://www.ibm.com/us/en/) also experienced a successful transition from a hardware manufacturer to the world’s largest information technology services company. They share a common ground of using service supply chain as their core management idea. Service supply chain, as the service-oriented integration supply chain, provides customers with complete set of products and integration services, which aims at achieving customer success and the efficiency running of whole supply chain. How to select the supplier portfolio from all over the world is extremely important and impacts on the competitiveness of the value-added networks, and ultimately decides the success or failure of the service supply chain [3].

Up to now, there has been three lines of work to address the problem of supplier portfolio selection, one is the multistage portfolio selection model, another is optimization method and the the third is the multi-features decision method. Specifically, multistage portfolio selection model decompose the problem into multistage and select supplier chains based on some arbitrary rules. For example, Shi et al. [29, 30] and Rajan et al. [24] studied supplier selection of two-stage, three-phase and four-stage model respectively. The second kind of methods conduct optimization method to select the suppliers portfolio, such as the analytic hierarchy process [26], data envelopment analysis [12], and fuzzy theory [33], genetic algorithms [31], hybrid genetic Particle Swarm algorithm [4] and hybrid intelligent algorithms [19]. The second method try to integrate multidimensional features or criteria to support the supplier selection in a supply chain [16, 20]. Although existing researchers have provided many rational approach methods for supplier selection, they failed to solve multiple supplier portfolio selection very well. Moreover, the existing methods do not meet the dynamics of multiple supplier portfolio selection because of the fact that the demands of different customers varies with different orders. Specifically, a supplier portfolio may satisfy the demand of a specific order for a customer, while not satisfy another order for the reason that different orders may have different features, such as demand quantity and order expiration etc. Rigorously, our method is related with all of the three methods. Specifically, our method is a 2-stage method which contains the filtering stage and re-ranking stage. Our method will optimize a loss function which minimizes a error ranking probabilistic function. Moreover, our method integrates multidimensional decision factors into the decision making process within the ranking neural network [7] which can learn experiences from the historical supply chain data.

In this paper, we propose an order-driven algorithm for supplier portfolio selection, which recommends different supplier portfolios for different orders dynamically, even for the same customer. The proposed model can learn from historical experiences intelligently, in this way the selection model will be smarter and smarter with the accumulation of order data for a service integration business. To this end, we formalize a large scale of features for suppliers, orders and customers. The ranking neural network is introduced to integrate the extracted features automatically. The weights for each features are automatically set by optimizing the decision gain, i.e., a quality of evaluation metric for a recommended list of supplier portfolio. Extensive experiments are conducted on a crowdsourcing data set, show that our method outperform the state of the art baselines. The simulation experiments also demonstrate the feasibility and effectiveness of the method.

Our work is also related to some other existing work. They can be categorized into three research areas, i.e., supplier selection [5, 10, 17, 36], information retrieval [9, 18, 21, 22, 25, 27] and machine learning [2, 13, 14, 23, 34]. Some latest literatures in the field of supplier selection, such as fuzzy AHP [5, 10], and a number of extended AHP models [17, 28, 36] are proposed in recent years. Fuzzy AHP based supplier selection methods can estimate the suppliers performance based on fussy multi-rules. The difference between ours and fussy-AHP methods is that we focus on selecting supplier portfolio for a given order, while fuzzy AHP models focus on estimating suppliers performance independent of orders. The core task of information retrieval is to rank documents in an appropriate way so that the search engine users feel relevant to their information need. The application scope of information retrieval is very spread, such as e-commerce system, recommendation system and news system etc. Our work borrows the idea in information retrieval and regards the supplier portfolio as document, which is natural and promising. We also borrow the nDCG@n [1] designed for information retrieval as the evaluation metric in this paper. The value of nDCG@n means the probability that the decision maker makes a right decision or select some proper supplier portfolios. In the portfolio selection method in this paper, we utilize the machine learning method. The core idea of machine learning is to learn experience from data. In our model, we will train a ranking neural network from the historical order data, which can rank portfolios according to the metric learned from experiences. Note that, all learning to rank algorithms can be used in the proposed portfolio selection framework. The reason we use the RankNet model is that it can learn a selection model based on the partial-order relationship among different supplier portfolios given specific orders.

In a nutshell, the contributions of this paper can be summarized as follows:

1. we define the novel service supply chain and clarify the intrinsic differences from the traditional supply chain;
2. we develop a novel supplier portfolio selection model based on the ranking neural network which can integrate multiple features automatically;
3. we conduct extensive simulation experiments on crowdsourcing data to demonstrate the feasibility and effectiveness of the proposed method.

The rest of the paper is organized as follows: (1) We introduce the basic concepts for service supply chain and neural network. Then, the overall supplier portfolio selection algorithm is introduced; (2) we summarize the decision features for supplier portfolio selection; (3) We formalize the training method of ranking neural network for supplier portfolio selection; (4) We conducted extensive simulation experiments on a collected crawdsourcing data; (5) We further demonstrate the possible applications of the proposed model; (6) We conclude this paper briefly and present the possible research direction for future work.

## Basic Concepts

In this section, we introduce the related basic concepts, such as the service supply chain and the ranking neural network. Our supplier portfolio selection model is designed for the application environment of service supply chain based on the ranking neural network.

### Service Supply Chain

**Fig 1:**
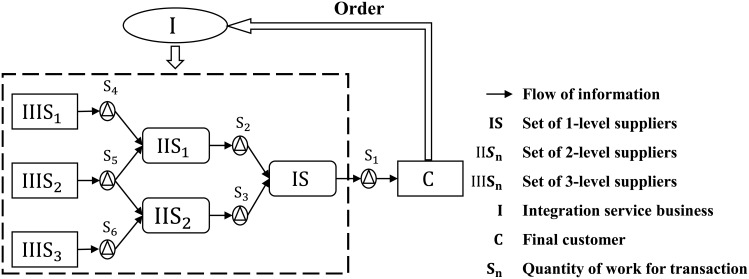
Conceptual graph for the service supply chain.

Based on the rich research results from all over the world [3, 11, 15, 35], we define the service supply chain as follows:

**Service Supply Chain** is a complex and customized value-added network structure for customers coordinated by a unified integration service business aiming at achieving customer success and the maximization of the whole supply chain value. Service supply chain is running driven by customers’ orders, which decomposes a customer’s order into multiple fine-grained steps (e.g., development, forecast, supply, production, distribution, retail and sale). The customized network includes a integrated logistics, information, capital, knowledge and any possible resources, which helps the customer formalize significant competition advantage over other competitors. In service supply chain, the service integrated business is in charge of the overall link of integration and full management for various service elements. The service supply chain have intrinsic differences from the traditional supply chain. The traditional supply chain is more arbitrary and has no a unified management service unit, i.e., the integration service business. Compared with the traditional supply chain, service supply chain is more intelligent and requires more dynamic decision making mechanism, especially for the supplier portfolio selection system. Fig 1 shows an example for service supply chain with 3-levels suppliers from 6 categories of suppliers.

Take the Fig 1 as an example, driven by customers’ order, the integration service business decomposes the order in stepwise and selects the optimized supplier portfolio from a global supplier pool then achieves the value-added network. Supplier portfolio selection problem in service supply chain and traditional manufacturing supply chain have some differences: (1) service supply chain selects vertical structure of suppliers ordered combination in global range (such as, raw materials suppliers, middle processing suppliers, assembled suppliers, distribution suppliers, and sales suppliers), while traditional supply chain select suppliers in a horizontal suppliers collection; (2) service supply chain elects supplier portfolio to achieve a completed customer order in the same time, while the traditional supply chain select suppliers for accomplishing a single process for an order; (3) the management method for suppliers in service supply chain differs from the traditional supply chain. Specifically, the former will require the suppliers reserve sufficient production ability, and will conduct a comprehensive supply chain planning and organization, while the traditional one will not need these operations. The supplier portfolio selection is more challenging than that in the traditional supply chain.

A service supply chain requires multiple suppliers, for example the chain in Fig 1 needs 6 suppliers. Each category of suppliers may have a large scale of candidate suppliers. Different suppliers may have some potential influences on each other and may have some constraints from other suppliers. For example, a supplier begin their work conditioned on the accomplishment of the work for the previous suppliers. Therefore, it is extremely difficult to select a superior supplier portfolio which can bring maximized gain for the whole supply chain.

### Ranking Neural Network

**Fig 2:**
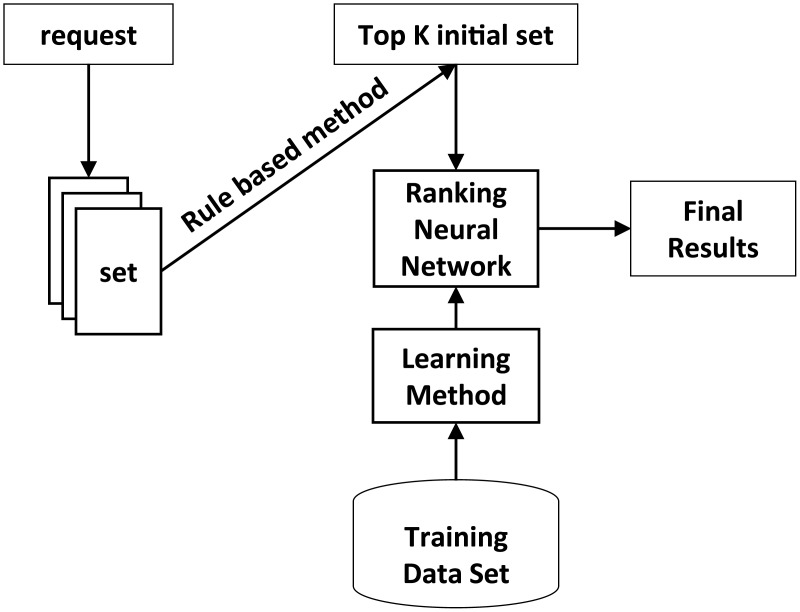
The work flow graph for the ranking neural network.

Ranking neural network [7] is based on the traditional BP neural network [6], which adopts the ranking error between sorted objects as the target loss function to train a special neural network using gradient descent algorithm [6] and is then applied to ranking objects considering multiple features. Fig 2 presents a simple work flow graph for the ranking ranking neural network. Ranking neural network inputs feature vector for all objects and output the ranking scores for a list of objects. Compared with traditional neural networks, ranking neural network can solve the supplier portfolio selection problem which depends on the specific order. The traditional method of supplier selection only consider the inherent properties of a particular supplier, and does not take into account the special requirements of a specific order. This article assumes that, quality of suppliers is relative, the advantages or disadvantages of supplier portfolios for different orders are not the same. For this reason, we cannot simply predict an optimal value (e.g., profit) to select suppliers. Ranking neural network considers the relative advantages and disadvantages of suppliers with respect to a specific order, while not directly use single indicator as a loss function. In this way, we can effectively select excellent supplier portfolio for a particular customer order.

## Supplier Portfolio Selection Algorithm

The supplier portfolio selection algorithm proposed in this paper is order-driven, which means that the algorithm will recommend to the service integration business with different supplier portfolios for different customer orders. The fundamental of this algorithm is to maintain supplier database which contains multiple categories of suppliers. Each category contains a large number of candidate suppliers. This article assumes that, before running the algorithm, the service integration business has accumulated a great deal of historical data. The data records the detailed information for each order including the supplier portfolio selection in fact and the corresponding overall benefit degrees (e.g., excellent, good or bad etc.) which are evaluated by the decision makers according the real profit of corresponding service supply chain.

To develop the algorithm we should first build a candidate set for supplier portfolio selection, in which each element is a supplier portfolio. Each supplier portfolio corresponds to a series of features. The candidate set is obtained by the Cartesian product of *C*_1_, *C*_2_, …, *C*_*N*_ as Eq 1 described:

$$\begin{array}{c} S=\{c_{i}:s_{i1}s_{i2}...s_{iN}|s_{i1}\in C_{1},s_{i2}\in C_{2},...,s_{iN}\in C_{N}\} \end{array}$$ (1)

where *S* represents the candidate set of supplier portfolios, *c*_*i*_ represents a supplier portfolio consisting of *N* categories of supplier, i.e., *s*_*i*1_ *s*_*i*2_…*s*_*iN*_. *s*_*iN*_ represents a supplier in category *C*_*j*_, where $1\leq i\leq \left|S\right|=\prod_{j=1}^{N}\left|C_{j}\right|$. Note that the inner sequence of suppliers in a portfolio is decided by the real need of an order, therefore we do not care the sequence of the inner suppliers. This candidate set is extremely large, so that it is impossible to select a optimal portfolio from this candidate set. Automatic selection method is needed.

**Fig 3:**
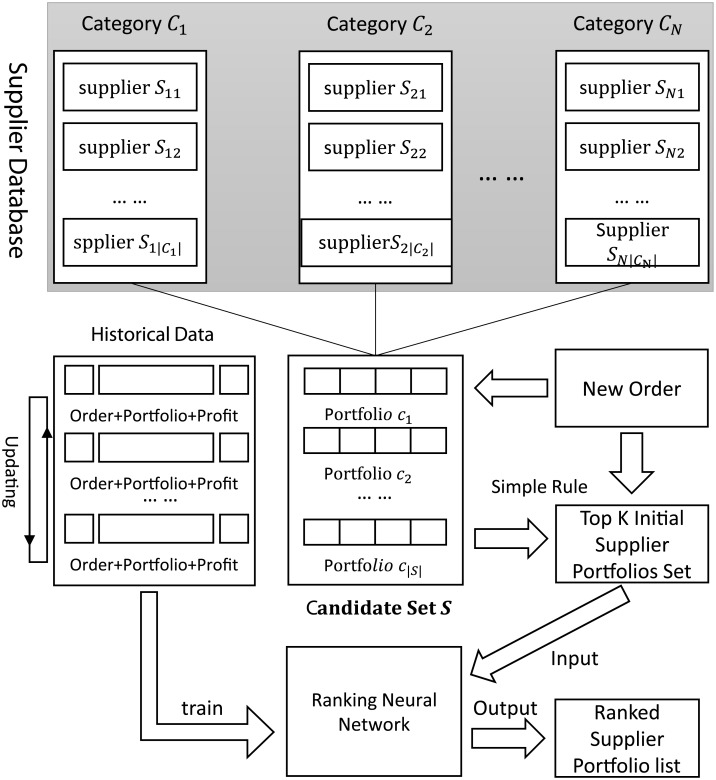
The framework of supplier portfolio selection algorithm.

In this paper, for an order request, the automatic algorithm will first filter out most of the supplier portfolios which do not meet the order requirements, and recommend several supplier portfolios as the initial candidate set. As the initial recommended candidate set is obtained by simple rules, the quality of portfolios in the set is not optimal. In order to select the optimal supplier portfolio, the ranking neural network will be adopted to re-rank the candidate set. The top-ranked supplier portfolio will be regarded as the best one and be recommended to the decision maker. Fig 3 illustrates the framework of supplier portfolio selection algorithm. The formalized algorithm is described in Algorithm 1. Specifically, the algorithm receives an input ordfer *o* and outputs a series of supplier portfolios as recommendation. To this end, we will maintain a supplier database containing *N* categories and category *C*_*i*_ contains |*C*_*i*_| candidate suppliers. Based on the database, we can build a candidate supplier portfolios set *S* which will be updated with the updating of supplier database. The algorithm will train a Ranking Neural Network (RNN) model from the historical order data, or update the model with the accumulation of historical data. When a new order is issued, the algorithm will first filter out the non-satisfied supplier portfolios according to some simple rules, and obtain a initial portfolio set *S*_*K*_ with *K* supplier portfolios. Then, extract feature vectors for each “order-portfolio” pair corresponding to each portfolio in *S*_*K*_. We can compute the model score for a portfolio by inputing its feature vector into the trained RNN model. Finally, we recommend the top *M* portfolios to the decision maker according to the computed model scores. Note that, after the accomplishment of this order, the decision maker needs to evaluate the profit degree of this order and store the data to the historical dataset. Accordingly, the RNN model should also be updated. The detailed training method for RNN will be described in specific section.

**Algorithm 1:** Supplier portfolio selection method.

1: INPUT: an order *o*;

2: OUTPUT: top *M* portfolios $P:\{c_{1},c_{2},...,c_{M}\}$;

3:P←ϕ;

4:$C\leftarrow \{C_{1},C_{2},...,C_{N}\}$; //*C*_*i*_ is a supplier set for category *i*.

5: *S* ← {*c*_*i*_: *s*_*i*1_ *s*_*i*2_…*s*_*iN*_|*s*_*i*1_ ∈ *C*_1_, *s*_*i*2_ ∈ *C*_2_, …, *s*_*iN*_ ∈ *C*_*N*_};

6: $D\leftarrow \{d_{1},d_{2},...,d_{\left|D\right|}|d_{i}=<o_{i},p_{i},profit_{i}>\}$; //D is the historical data set.

7: Learning $M_{RNN}$ from D; // $M_{RNN}$ is a Ranking Neural Network model.

8: *S*_*K*_ ← {*c*_1_, *c*_2_, …, *c*_*K*_}; //*c*_*i*_ is a portfolio satisfiying some simple rules.

9: $F_{K}\leftarrow \left\{F_{1},F_{2},...,F_{K}\right\}$; // *F*_*i*_ is a feature vector for a “order-portfolio” pair “*o*-*c*_*i*_”.

10: **for** *i* = 0 to *K* **do**

11:  $Score_{i}\leftarrow M_{RNN}\left(F_{i}\right)$;

12: **end for**

13: $P\leftarrow \{c_{1},c_{2},...,c_{M}\}$; //Select top *M* portfolios according to *Score*_*i*_ of *c*_*i*_.

14: RETURN P;

## Feature Extraction

In the environment of service supply chain, the selection of supplier portfolios is subject to various complex factors. Decision makers of business expect to find some mechanisms find superior supplier portfolios under the constraints of different factors and to achieve purpose of benefit optimization. The criteria for selecting supplier portfolios vary with different orders. In other words, the selection of supplier portfolios should not only depend on their own strength, but also take into account the relationship between the order and corresponding suppliers. In order to achieve the purpose of benefit optimization, we formalize a large number of factors. Moreover, the relationship characteristics between order each candidate supplier are also integrated into the feature vector, denoted as *x* ∈ *R*^*n*^. Each element in the vector representing a feature corresponds to a decision factor. All features can be classified into three categories: (i) features of suppliers; (ii) features of the order; (iii) features of order-supplier relationships. Therefore, the proposed supplier portfolio selection model can be seen as a dynamic model which considers multiple order-specific features. We will present the three categories of features respectively and interpret their meaning in detail.

### Features of Suppliers

**Fig 4:**
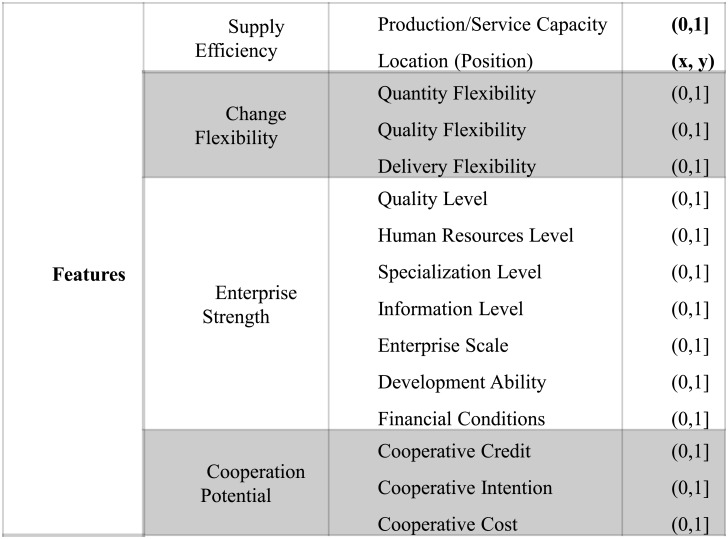
Features of supplier.

We propose a systematic evaluation mechanism for suppliers which is presented in Fig 4. The third column is the range of corresponding feature value.

#### Supply efficiency

The supply efficiency of suppliers is impact by their productivity and geographic location, which is an important factor for supplier portfolio selection. General speaking, most orders need timeliness, which requires the selected suppliers have appropriate productivity (in this paper, the requirement of productivity for an order should account for 30%–70% of the total productivity of a supplier), since that lower productivity of suppliers means that suppliers cannot provide production in time, while too high productivity of suppliers may result in that the supplier will not put sufficient emphasis on this order. Certainly, other intervals can also be used according to the requirement of the service supply chain. To some extent, the location of suppliers affect the speed and cost of logistics. In the service supply chain, the product of suppliers at a higher level will be delivered to the suppliers at next level. Similarly, the product of final suppliers will be delivered to the final customer directly. Therefore, the distance between subsequent suppliers determines the speed and cost of logistics. In an ideal situation, if the productivity of a supplier is satisfied, the distance factor will be a essential factor to be considered by the decision makers. In practice, distance between the suppliers is impacted by complex factors, e.g., local traffic conditions, terrain and weather etc. For the sake of computing simplicity, in this paper, we adopt the simple Euclidean distance as the distance between suppliers. To this end, each supplier corresponds to a location in the two-dimensional coordinates *p*: (*x*, *y*).

#### Change flexibility

In general, a integration business prefer flexible suppliers, because greater change flexibility means smaller cost of order alteration. In this study, we assume that an order may have some possibility to change someway in the process, but the possibility is different for different orders. For the orders with larger change probability, it has rigorous requirement of flexibility for suppliers. While for the orders with small change probability, the requirement of flexibility for suppliers is also small. However, in fact, we need an appropriate change flexibility for suppliers which is dependent on specific orders.

#### Enterprise Strength

Intuitively, integration business tend to select suppliers with appropriate strength to cooperate. It is not always good to select a extremely strong enterprise, because the big enterprises may require relatively higher cost, and the willingness to cooperate may be not positive. The enterprise strength should also be adapted to specific orders. Specifically, when the orders’ scale is large, we should select relatively strong supplier, which guarantee that the orders be completed on time with high quality, and vise versa.

#### Cooperation Potential

We can evaluate the cooperation potential according to the historical order data and cooperation experiences with the suppliers. The cooperative credit, willingness to cooperate and cooperative cost are three important factors which affect the potential for cooperation.

### Features of the Order

Service supply chain is driven by order, the integration business select optimal supplier portfolio to build a competitive value-added supply chain.

Customer orders include the following features: (i) Capacity of product: after decomposing the customer orders into several levels, we should determine required capacity of product for a supplier in specific level. The reservation productivity should be controlled in the interval of 30%–70% (we set this interval, since that lower productivity of suppliers means that suppliers cannot provide production in time, while too high productivity of suppliers may result in that the supplier will not put sufficient emphasis on this order. Certainly, other intervals can also be used according to the requirement of the service supply chain.). In this way, we can ensure that the selected supplier will put sufficient attention to this order; (ii) Customer location: customer’s location has a great influence on supplier portfolio selection; (iii) Lead time: the leading time required by customer; (iv) Order flexibility: the possibility of order change, product change, volume change, and lead change; (v) Order quotas: integration business should first decompose the orders and determine quotas for different levels of suppliers.

### Features of “order-portfolio” Relationship

In order to select the order-specific supplier portfolio, each pair of “order-portfolio” is represented as a normalized feature vector. Each dimension of the vector represents a characteristic impacting the selection of supplier portfolio. The relationship features between order and suppliers (for example, productivity, distance and flexibility etc.) are computed automatically according to the intrinsic features of suppliers and orders, which will be formalized as follows:

**Productivity Ratio:** We use the productivity ratio (The ratio of order productivity requirements accounting for the total productivity of a certain supplier) to measure productivity relationship between the order with a supplier. This feature is formalized as follows:

$$\begin{array}{c} R_{os}^{C_{i}}=\frac{P_{o}^{C_{i}}}{P_{s}^{C_{i}}} \end{array}$$ (2)

where $R_{os}^{C_{i}}$ represents the relationship between order *o* and supplier *s*, $P_{o}^{C_{i}}$ represents the productivity requirement of the order suppliers in class *C*_*i*_. $P_{s}^{C_{i}}$ is the productivity of supplier *s* in class *C*_*i*_. For a specific order, each supplier corresponds to a productivity ratio feature.

**Distance:** To some extent, the distance between customer and suppliers, and the distance between suppliers at different levels determine the cost of logistics. We expect to select supplier portfolio which can guarantee the minimization of the total distance. We define *D*_*o*_ *c* as a distance measurement of between order and a supplier portfolio. This relationship feature is formalized as follows:

$$\begin{array}{c} D_{oc}=\sum_{s_{a}\in C_{III},s_{b}\in C_{II}}D\left(s_{a},s_{b}\right)+\sum_{s_{a}\in C_{II}}D\left(s_{a},S_{I}\right)+D\left(S_{I},customer\right) \end{array}$$ (3)

where *C*_*III*_ and *C*_*II*_ represent the suppliers in level *III* and *II*, *S*_*I*_ represents the level I supplier (see Fig 1). *D*(*s*_*a*_, *s*_*b*_) represents the Euclidean distance between two suppliers. This feature will be normalized by Eq $nD_{oc}=D_{oc}/D_{oc}^{max}$, where $D_{oc}^{max}$ is the maximized distance in all candidate supplier portfolios.

**Flexibility:** this feature is co-determined by the order flexibility and the supplier flexibility, which is formalized as follows:

$$\begin{array}{c} F_{os}^{f}=p_{o}^{f}\times F_{s}^{f} \end{array}$$ (4)

where $F_{os}^{f}$ represents the flexibility value of supplier *s* for the order *o* with respect to the flexibility *f* ∈ {*quantityflexibility*, *qualityflexility*, *expectationflexibility*}, $p_{o}^{f}$ and $F_{s}^{f}$ are the flexibility of order and supplier with respect to *f*.

### Normalization of Feature Vector

We have formalized feature vector with 85 dimensions including all order features, supplier features and the relationship features. For the compatibility between different feature vectors, we normalize the feature vector by the widely used “min-max” normalization method, which is formalized as follows:

$$\begin{array}{c} normF_{i}=\frac{F_{i}-minF_{i}}{maxF_{i}-minF_{i}} \end{array}$$ (5)

where *F*_*i*_ is a feature dimension, *minF*_*i*_ and *maxF*_*i*_ are the minimized and maximized feature value corresponding to the dimension *i*. The normalization process will guarantee the feature in the interval [0, 1].

## Ranking Neural Network for Supplier Portfolio Selection

In this paper, we train a Ranking Neural Network (RankNet) [7, 8] from the historical order data. Specifically, we need to assign a label for each historical “order-portfolio” pair to indicate the appropriateness. Note that when we evaluate a supplier portfolio for an order, the corresponding supply chain is finished. The label is selected from (4, 3, 2, 1, 0), the number is proportional to the appropriateness of a supplier portfolio for a specific order. For example, for an order *o*_1_, a supplier portfolio *c*_1_: *abc* is labeled as 4, and another supplier portfolio *c*_2_: *abd* is labeled as 2. This shows that *c*_1_ better suited to order 1 than *c*_2_. A large scale of labeled order data can be used to train a ranking neural network which can recommend good supplier portfolio to the decision maker. In rest of this section, we introduce the training process of ranking neural network (RankNet) in detail.

**Fig 5:**
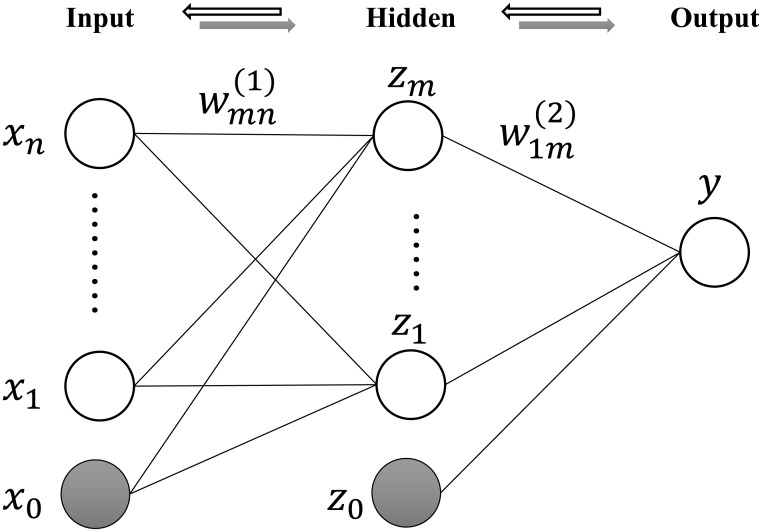
Ranking Neural Network.

In this paper, we define a neural network which contains three layers, i.e., the input layer, hidden layer and the output layer (see Fig 5). The input layer is the “order-portfolio” pair feature vector. Each node is a neuron which corresponds to a feature dimension. The output layer only has one neuron which is the ranking value defined as *y* = *f*(*x*).

$$\begin{array}{c} f\left(x\right)=\sigma (\sum_{j=1}^{m}w_{j}^{\left(2\right)}\sigma \left(\sum_{i=1}^{n}w_{ji}^{\left(1\right)}x_{i}+w_{j0}^{\left(1\right)}\right)+w_{0}^{\left(2\right)}) \end{array}$$ (6)

where *σ*(*a*) is a active function: $\sigma \left(a\right)=\frac{1}{1+\exp(-a)}$, *w*^(1)^ and *w*^(2)^ are the weights which are need to be estimated.

### Ranking Function

### Loss Function

For the reason that the label of each supplier portfolio is a integer between 0 and 4, while the output of the ranking neural network is a float between 0 and 1, which leads to that the label value and the output value is not in the same measurement space. Therefore, it is not appropriate to use the traditional least square function as loss function to train the ranking neural network. To address this problem, Burges et al. proposed a probabilistic loss function to substitute the traditional least square function [6, 7]. In this paper, we use this method to solve the supplier portfolio selection problem. In this method, we only consider the relative ranking between two candidate supplier portfolios rather their absolute evaluation values. In this way, we can obtain a ranked list of candidate supplier portfolios. Because the neural network only care rank of the candidate supplier portfolios, therefore we call it ranking neural network, which is abbreviated with RankNet.

Given an order, two supplier portfolios *c*_*i*_ and *c*_*j*_ are represented with feature vectors *x*_*i*_ and *x*_*j*_, the corresponding output values of the RankNet are *s*_*i*_ = *f*(*x*_*i*_) and *s*_*j*_ = *f*(*x*_*j*_) respectively. The output value of RankNet can be mapped into a posterior probabilistic which indicates the probability that the *c*_*i*_ is better than *c*_*j*_ by a logistic function:

$$\begin{array}{c} P_{ij}\equiv P\left(c_{i}⊳c_{j}\right)\equiv \frac{e^{\left(s_{i}-s_{j}\right)}}{1+e^{\left(s_{i}-s_{j}\right)}} \end{array}$$ (7)

where *c*_*i*_⊳*c*_*j*_ is the event that *c*_*i*_ is better than *c*_*j*_. We use ${\bar{P}}_{ij}$ to indicate the theoretical best value, namely the prior probability that *c*_*i*_ is better than *c*_*j*_ mining from the historical data.

$$\begin{array}{c} {\bar{P}}_{ij}\equiv \frac{1}{2}\left(1+S_{ij}\right) \end{array}$$ (8)

where *S*_*ij*_ ∈ {0, ±1}. Specifically, if *c*_*i*_ is better than *c*_*j*_ *S*_*ij*_ = 1, if *c*_*i*_ is worse than *c*_*j*_ *S*_*ij*_ = −1, else *S*_*ij*_ = 0. Therefore, ${\bar{P}}_{ij}\in \left\{0,0.5,1\right\}$.

The output probability *P*_*ij*_ has some errors compared with the theoretical prior probability ${\bar{P}}_{ij}$. The cross entropy between the output probability distribution and the prior probability distribution is utilized as the loss function (the error of the ranking model), which is formalized as follows:

$$\begin{array}{c} C_{ij}=-{\bar{P}}_{ij}\log P_{ij}-\left(1-{\bar{P}}_{ij}\right)\log\left(1-P_{ij}\right) \end{array}$$ (9)

Substitute Eqs 7 and 8 into Eq 9, we obtain following equation:

$$\begin{array}{c} C_{ij}=-\frac{1}{2}\left(1+S_{ij}\right)\left(s_{i}-s_{j}\right)+\log\left(1+e^{s_{i}-s_{j}}\right) \end{array}$$ (10)

Eq 11 is the final loss function, based on which will train the ranking neural network for supplier portfolio selection.

### Training

We compute the partial derivative of *C*_*ij*_ corresponding to *s*_*i*_ and *s*_*j*_ respectively.

$$\begin{array}{c} \frac{\partial C_{ij}}{\partial s_{i}}=-\frac{1}{2}\left(1+S_{ij}\right)+\frac{e^{s_{i}-s_{j}}}{1+e^{s_{i}-s_{j}}}=-\frac{\partial C_{ij}}{\partial s_{j}} \end{array}$$ (11)

We adjust the weights *w*_*k*_ ∈ *R* in the network along the direction of gradient with a learning rate *η* (we set *η* = 0.00005 in this paper) to decrease the value of loss function. The adjust method is formalized as follows:

$$\begin{array}{c} w_{k}\leftarrow w_{k}-\eta \frac{\partial C_{ij}}{\partial w_{k}}=w_{k}-\eta \left(\frac{\partial C_{ij}}{\partial s_{i}}\frac{\partial s_{i}}{\partial w_{k}}+\frac{\partial C_{ij}}{\partial s_{j}}\frac{\partial s_{j}}{\partial w_{k}}\right) \end{array}$$ (12)

The change value (Δ) of the loss function after updating can be computed as Eq 13:

$$\begin{array}{c} \Delta C_{ij}=\sum_{k}\frac{\partial C_{ij}}{\partial w_{k}}\Delta w_{k}=\sum_{k}\frac{\partial C_{ij}}{\partial w_{k}}\left(-\eta \frac{\partial C_{ij}}{\partial w_{k}}\right)=-\eta \sum_{k}{\left(\frac{\partial C_{ij}}{\partial w_{k}}\right)}^{2} \end{array}$$ (13)

Δ*C*_*ij*_ is a negative value, which shows that after updating by Eq 12 the loss function will be definitely decreased. In this way, the loss function will reach a convergence after sufficient iterations.

For any future order, we obtain a ranked list of supplier portfolios according to the output values of the RankNet for supplier portfolios. The top-ranked supplier portfolio will be recommended to the decision maker of the service supply chain.

## Empirical Evaluation

We propose a novel supplier portfolio selection method for the service supply chain. In this section, we conduct extensive experiments on a crowdsourcing data, which aim at demonstrating the feasibility and effectiveness of the proposed method. In the following, we first introduce the crowdsourcing data, then introduce the evaluation metric and finally report the experimental results.

### Crowdsourcing Data

We gather evaluation data through Amazon Mechanical Turk (AMT) which is a “crowdsourcing” platform for exploiting the wisdom of collective intelligence. We first design some orders and suppliers, then generate a series of supplier portfolios for each order automatically, and then publish them to the AMT, AMT will send them to specific users and ask users to complete our tasks. The tasks require AMT users to label the expected profit degree of each supplier portfolio given a specific order. We can supervise the labeling process through the AMT interface. We require that each supplier portfolio must receive 3 users’ valid labels. The average value of 3 labels is used as the final label of the portfolio. In this way, we can obtain a number of reliable “order-portfolio-label” data, which can reflect the value of each portfolio for specific order to some extent.

Specifically, we design 6 categories of suppliers according to Fig 1. Each category contains 10 suppliers. We design 100 orders, each order follows 50 supplier portfolios which are filtered by some simple rules (e.g., productivity ratio, distance, cooperative intention etc.). Each supplier portfolio will be labeled by the AMT user with a integer between 0 and 4 which indicates the appropriateness (expected profit degree) corresponding to a specific order. We only use the high-qualification turkers whose HIT Approval Rate (%) ≥ 95. We filtered out the data of turkers whose dwell time was less than 30s for each portfolio.

**Fig 6:**
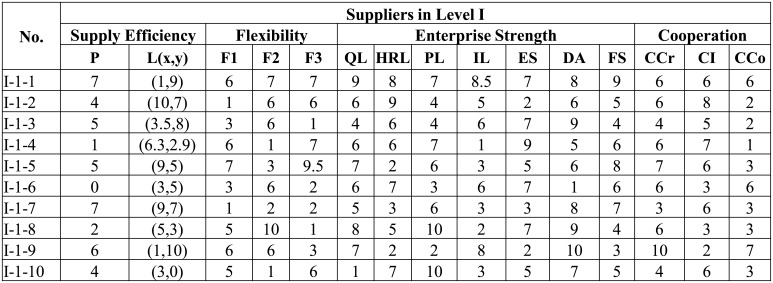
Examples of suppliers in level I. For the limitation of the space, we use some abbreviations. They are, P: Productivity, L: Location, F1: Quantity Flexibility, F2: Quality Flexibility, F3: Expectation Flexibility, QL: Quality Level, HRL: Human Resources Level, PL: Professional Level, IL: Informational Level, ES: Enterprize Scale, DA: Development Ability, FS: Financial State, CCr: Cooperative Credit, CI: Cooperative Intention, CCo: Cooperation Cost. Detailed data can be found in the supporting information (S1 Suppliers and Labeled Orders).

**Fig 7:**
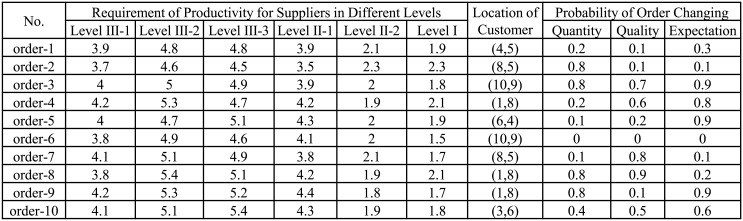
Examples of the designed orders. Each line shows the data example for some specific features. The features will be processed by our feature extraction tool programmed with Java programming language. Detailed data can be found in the supporting information (S1 Suppliers and Labeled Orders).

**Fig 8:**
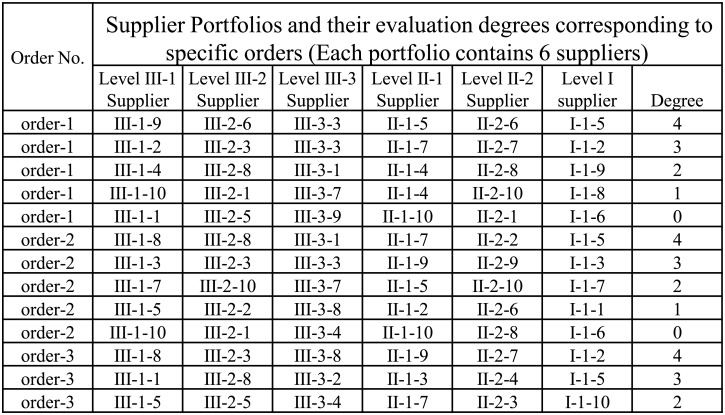
Examples of the labeled suppliers portfolios. Each line indicates a supplier portfolio which contains six suppliers in different categories. The last column is the degree which indicates the degree of each portfolio evaluated by the volunteers according to the possible profit for specific orders. Detailed data can be found in the supporting information (S1 Suppliers and Labeled Orders).

Fig 6 shows some examples of designed suppliers in level I. We do not show examples for other 5 categories, since they are in the same format to the the category Level I. Similarly, we also show some examples for the designed orders in Fig 7. Some examples of supplier portfolios are presented in Fig 8, where each supplier portfolio contains 6 suppliers selected from 6 categories respectively.

### Evaluation Metric

For each tested order, we recommend a ranked list with 10 supplier portfolios to the decision maker. A decision maker needs further decide which supplier portfolio to be selected. Intuitively, the top ranked supplier portfolio will have more probability to be selected. Therefore, if a better portfolio is ranked higher than a worse portfolio, the decision maker will have more probability to do a right decision, which leads to better gain for the whole service supply chain. In order to measure the quality of the recommendation list of supplier portfolios, we adopt the DCG (Discount Cumulative Gain) [8] as the evaluation metric, which is defined as follows:

$$\begin{array}{c} DCG@n=\sum_{i=1}^{n}\frac{2^{r_{i}}-1}{\log(1+i)} \end{array}$$ (14)

where *n* is the number of portfolios in the ranked list (*n* ∈ {1, 2, 5, 10} in this paper), *r*_*i*_ ∈ {0, 1, 2, 3, 4} is the degree of the supplier portfolio ranked at *i*, the degree is manually labeled. The more *r*_*i*_, the better the portfolio is. DCG awards the superior portfolios ranked higher and penalizes the lower ranked portfolios. Overall, the larger the *DCG*@*n* value of the recommendation list, the better the quality of the list will be. In order to map the *DCG*@*n* into the interval [0, 1], we normalize it by following equation:

$$\begin{array}{c} nDCG@n=\frac{DCG@n}{idealDCG@n} \end{array}$$ (15)

where *nDCG*@*n* is the abbreviation for Normalized Distinct Cumulative Gain, *idealDCG*@*n* is the *DCG*@*n* value of the recommendation portfolio list at the ideal rank (best rank from top to last according to the appropriate degrees of portfolios). In this paper, we report the simulation results with *nDCG*@*n*.

We adopt the *nDCG*@*n* designed for information retrieval as our evaluation metric based on a decision making assumption. We assume that a decision maker will select the final supplier portfolio from a set of possible supplier portfolios. The *n* value is the size of the possible set. The value of *nDCG*@*n* means the possibility that the decision maker can make a right decision. The more *nDCG*@*n* value, the more possibility of making a right decision. Intrinsically, the process of decision making is also a process of information retrieval.

### Experiment Setup

Three comparative models are tested on the same crowdsourcing data described in the previous section. They are listed as follows:

1. RBM (baseline), is the Rule Based Method, which recommends a list of supplier portfolios according to some simple rules presented in previous feature extraction section, such as the productivity ratio is 30%–70%, the distance is as near as possible, the reservation ratio for an order is 30%–70% and so on.
2. TNN (baseline), is the Traditional Neural Network, which predicts the appropriate degrees for the candidate supplier portfolio directly with least square method (as known as the BP neural network) [6].
3. RNN, is the Ranking Neural Network proposed in this paper, which ranks the supplier portfolio list by pairwise method.

Five-folds cross validation are conducted for TNN and RNN. Specifically, we segment the designed 100 orders into 5 groups, each group has 20 orders. For each fold, one group of orders are utilized as testing data, another group as the validation data and the rest of the groups as the training data. The training set and validation set can be seen as the historical order data, the testing data can be seen as the future data. After running 5 folds, all orders will be tested. The average *nDCG*@*n* for all orders is regarded as the evaluation result for corresponding models.

### Results and Discussion

We tested three selection models with respect to *nDCG*@1, *nDCG*@2, *nDCG*@5 and *nDCG*@10. *nDCG*@1 simulates the condition that the decision maker select the top one supplier portfolio directly, similarly *nDCG*@2, *nDCG*@2 and *nDCG*@2 simulate that selecting one from top 2, 5 and 10 supplier portfolios. In the Table 1, significant Test (t-test) has been done for TNN and RNN compared with RBM, where the symbol ‡ means *p* < 0.01 with paired t-test, † means *p* < 0.05.

**Table 1:** Results for the simulation experiments.

Models	NDCG@1	NDCG@2	NDCG@5	NDCG@10
RBM	0.3563	0.3775	0.3849	0.3916
TNN	0.3629†	0.3992†	0.4037†	0.4172†
RNN	0.3923‡	0.4141‡	0.4248‡	0.4426‡

Table notes: The experimental results can be obtained from supporting information (S1 Suppliers and Labeled Orders). For the significant test, the symbol ‡ means *p* < 0.01 with paired t-test, † means *p* < 0.05.

Table 1 shows that both neural network based supplier portfolio selection models (TNN and RNN) outperform RBM significantly with respect to all evaluation metrics. Specifically, the traditional neural network is better than RBM, which shows that Machine Learning method can improve the selection performance. The reason is that TNN selects supplier portfolios based on the historical order data, which makes the selection model intelligent. Moreover, the proposed RNN model in this paper, further improves the selection performance significantly compared with TNN, which shows that Ranking Neural Network can learn more information from the historical data.

From the angle of portfolio size of the recommendation list, we find that the performance increases with the increase of the portfolio number. The possible reason is that recommending more reliable supplier portfolios can improve the average gain for the whole service supply chain. However, this may be unreasonable to some extent for the reason that more recommendation portfolios may lead to the decision dilemma. This dilemma makes the decision maker difficult to decide which portfolio to select.

### Efficiency Analysis

The proposed supplier portfolio selection approach based on Ranking Neural Network (RNN) has the same complexity to the Traditional Neural Network (TNN) [8], since that the training method of RNN is based on the TNN. Therefore, RNN based approach can improve the recommendation performance compared TNN based approach without sacrificing the efficiency. Certainly, the Rule Based Model (RBM) is the most efficient model, since that it returns supplier portfolios based on simple rules and does not require any training process which is time consuming. However, RBM’s performance is poor, because it can not capture the complex relationships among diversified decision features in orders and suppliers of different levels.

## Possible Applications

The above experiments are conducted on the crowdsourcing data which has the same format to the corporation data. Therefore, the proposed supplier portfolio selection framework can be easily applied in real corporations which need select supplier portfolios from a large space of suppliers.

To apply the proposed supplier portfolio selection model, we should first design a database which can store all of the required information including the basic information of each suppliers, the detailed order information and the decision making information, etc. The database can be accessed and updated easily and efficiently. The designed database should also be extensible.

We should develop a special engine in charging of generating candidate supplier portfolios given each specific order and extracting the complex features for the pairs of order and candidate supplier portfolios. The engine is running online and can answer all online request for generating portfolios and extracting features.

A training engine should be developed. This engine is in charging of training and updating an ranking neural network. The training engine is triggered in two modes, i.e., regularly and order event. The first mode requires the training engine running regularly, e.g., each month. The order event mode requires the training engine starts to run when a new order completes. Two methods have their own advantages and disadvantages. For example, the first mode may lead to that supplier portfolio selection model can not response to the change of the application environment timely, while the second mode may lead to the problem of system overloading. We can find some strategies to balance the two modes. This is problem will be studied systematically in the future. The online learning theory can be used to guide the implementation of the training engine.

The supplier portfolio selection engine is developed to provide decision making suggestions. Each suggestion is a supplier portfolio. The engine will provide *K* supplier portfolios to the decision maker ordered by the values computed by the ranking neural network. *K* is a free parameter which can be set freely through a user interface.

## Conclusions and Future Work

In this paper, we proposed a novel supplier portfolio selection model for the service supply chain. To this end, we summarized a series of decision factors and then formalized a feature vector which contains the supplier intrinsic features, order intrinsic features and the order-portfolio relation ship features. The problem of portfolio selection are transformed into a ranking problem. The ranking neural network is utilized to rank the candidate supplier portfolios, which can learn experience from the historical order data intelligently. Extensive experiments are conducted on the crowdsourcing data, which demonstrated that the feasibility and effectiveness of the proposed method. We introduce the possible application method in real corporations for the proposed model.

However, we did not apply our method in a practical environment, such as a real service supply chain, which makes the practical supplier portfolio selection still a open problem. In the future, we will cooperate with the industrial community and develop a more practical supplier portfolio selection method for the service supply chain.

## Supporting Information

S1 Suppliers and Labeled Orders The data for all suppliers in different levels and for labeled orders with different supplier portfolios. In the compressed file S1_Suppliers_and_Labeled_Orders.zip, we can find 7 text files which are the needed data for our experiments. “S1_File_orders.txt” stores the labeled orders. “S1_File_suppliers-I.txt” stores the supplier information in Level I. Similarly, “S1_File_suppliers-II-1.txt, S1_File_suppliers-II-2.txt, S1_File_suppliers-III-1.txt, S1_File_suppliers-III-2.txt, S1_File_suppliers-III-3.txt” are the supplier information for specific categories.
(ZIP)
